# Antibiotic Prescribing Patterns for Patients with Pharyngitis in Malaysian Public Primary Care Clinics

**DOI:** 10.21315/mjms2022.29.1.9

**Published:** 2022-02-23

**Authors:** AbdulRahman Muthanna, Siti Zulaikha Zakariah, Aneesa Abdul Rashid, Sazlina Shariff Ghazali, Rukman Awang Hamat, Maliza Mawardi, Hani Syahida Salim, Nurainul Hana Shamsuddin

**Affiliations:** 1 Department of Medical Microbiology, Faculty of Medicine and Health Sciences, Universiti Putra Malaysia, Selangor, Malaysia; 2 Department of Biomedical Sciences, Faculty of Medicine and Health Sciences, Universiti Putra Malaysia, Selangor, Malaysia; 3 Department of Family Medicine, Faculty of Medicine and Health Sciences, Universiti Putra Malaysia, Selangor, Malaysia

**Keywords:** pharyngitis, primary care, antibiotics, prescription, inappropriate prescribing

## Abstract

**Background:**

Over-prescription of antibiotics for upper respiratory tract infection (URTI) is a continuing problem in Malaysia, leading to increased antimicrobial resistance and unnecessary cost incurred for treatment. In a patient presenting with a sore throat, it is recommended to only prescribe antibiotics to group A streptococcus (GAS) pharyngitis confirmed by a throat culture, rapid antigen test or in patients with a Centor score of 4.

**Methods:**

This cross-sectional study assessed the proportion of antibiotics prescribed and antimicrobial susceptibility patterns of GAS pharyngitis in the Malaysian primary care setting. Two-hundred and fifteen adult patients presenting with sore throat were recruited in three primary care clinics. Demographic data and clinical information were collected and analysed. Centor scores were calculated according to the clinical information and throat swabs were collected from all participants for GAS identification.

**Results:**

Only six throat swabs isolated GAS and indicated for antimicrobial treatment (2.8%). However, 48 participants (22.3%) were prescribed antibiotics out of which only four (8.3%) patients with isolated GAS, including three (6.2%) patients who clinically had a Centor score of 4 and one patient with a score of 3. Amoxicillin and erythromycin were the most commonly prescribed antibiotics (58.3% and 25% of all antibiotics, respectively).

**Conclusion:**

There is a high proportion of antibiotic prescriptions which were not indicated in patients with sore throat in this study. This may reflect a common practice of antibiotic overuse for sore throat in primary care settings in Malaysia. Concerted interventions to reduce the inappropriate prescribing of antibiotics are urgently needed.

## Introduction

Upper respiratory tract infection (URTI) is the most common acute illness seen in primary care clinics globally. In 2015, the global incidence of URTI was over 17 billion (1). URTI in adults are mainly caused by a virus; only about 10% of incidences are bacterial, mainly caused by group A streptococci (GAS), which is the only indication for antimicrobial therapy (2). In GAS pharyngitis, oral penicillin V for 10 days or a single dose of parenteral penicillin G effectively reduces symptom duration by 1 day to 2 days, spread of the disease, and incidence of toxigenic and immunologic complications (3).

In general, antibiotics are safe and provide moderate clinical benefits in a minority of patients with pharyngitis (4). Whereas, antibiotics should be prescribed only for patients with GAS pharyngitis (2). However, previous studies have reported that there is an over-prescription of antibiotics for URTI in general practice, where 73% of patients with pharyngitis were prescribed antibiotics (5, 6). In Malaysia, antibiotics are commonly prescribed by primary care providers and most frequently for respiratory tract infections (7).

Indiscriminate use of antibiotics has been identified as one of the major factors associated with the escalating rates of antimicrobial resistance (8). Also, it causes adverse effects (e.g. allergy or diarrhoea) and adds to the economic burden of the health care system worldwide (9).

GAS (*Streptococcus pyogenes*) and other beta-haemolytic streptococci are sensitive to penicillin and cephalosporin as well as rifampin and vancomycin (10, 11). However, some strains of the bacterium have been found to be resistant to macrolides, tetracycline and clindamycin (10). The present study determines the antibiotic prescribing rates and factors contributing to antibiotic prescription for adults with a sore throat in three primary care clinics in Malaysia. It extends to study the antimicrobial susceptibility patterns of beta-haemolytic streptococci (groups A, B, C, F and G) that cause pharyngitis and aims to determine if the antibiotics chosen for these conditions are consistent with the local antibiotic guideline (12).

## Methods

Using a cross-sectional study design, questionnaires containing demographic data, clinical information and treatment prescribed were administered and throat swabs were collected from ≥ 18 years old patients who complain of a sore throat. Patients were recruited using simple convenience sampling in three health clinics in Selangor, Malaysia. Patients who treated with antibiotics within two weeks, pregnant, very ill or who were immunocompromised (except those with diabetes mellitus) were excluded.

The sample size was calculated using single proportion sampling (13), based on the previous prevalence of pharyngitis in Thailand (14), with consideration of an estimated 20% incomplete data. The total sample size was 215.

Researchers screened patients at the triage counter of the clinics. Patient information sheets were provided to eligible patients. After reviewing the information and agreeing to participate, the patient would be administered an informed consent form and the researchers completed the study questionnaire. The researchers then collected a throat swab and later verified and completed the remaining clinical information (diagnosis and prescription) in the questionnaire according to the medical records.

The throat swabs were then cultured to identify β-haemolytic streptococci. Each β-haemolytic streptococci isolates further tested to identify streptococcal groups A, B, C, F and G. Subsequently, antimicrobial susceptibility testing according to the Clinical and Laboratory Standards Institute (15), were performed to study antibiotic susceptibility patterns of the pathogen against the following antibiotics: penicillin G, ampicillin, erythromycin, clindamycin, ofloxacin, cefepime, cefotaxime, ceftriaxone, tetracycline, linezolid and vancomycin.

### Data Analysis

Continuous data were presented as means and standard deviations. Categorical variables were presented as whole numbers and percentages. The comparison of categorical variables was made using Pearson Chi-squared and Fisher’s exact tests as appropriate. A probability of *P* < 0.05 was considered statistically significant.

## Results

### Demographic Characteristics

The total number of participants was 215 in the three clinics (response rate was 89.9%). Twenty-two patients were ineligible (five were treated with antibiotics within less than 2 weeks, two pregnant women and 15 were aged less than 18 years old), and two patients refused to participate in the study. The mean age was 36.43 years old. The proportion of female participants (57.7%) was slightly higher than male participants (42.3%). The majority of participants were Malay (62.8%) followed by Indian (30.2%), Chinese (5.1%) and others (1.9%).

### Antibiotic Prescription Rate

Antibiotics were prescribed in 48 (22.3%) out of 215 participants. Table 1 shows the sociodemographic characteristics of patients prescribed antibiotics. More male patients were given antibiotics in the clinics compared with female patients. Antibiotics were mostly prescribed to patients aged 18 years old–28 years old and Malay patients. Chi-squared test and Fisher’s exact test showed that there was a significant association between antibiotic prescription with ethnicity and smoking status (*P* < 0.05). Overall, cough and rhinorrhea are the most common clinical manifestations for antibiotics prescribed. Prescribed antibiotics were significantly associated with swollen anterior cervical lymph, tonsillar swelling or exudates pharyngitis/tonsillitis and non-GAS pharyngitis. Among 48 prescribed antibiotics, 3 (6.2 %) with Centor score 4, 6 (12.5%) with Centor score 2, 3 (6.3%) with Centor score 3 and 36 (75.0%) with Centor score 0–1. The results show that the Centor score of 0–1 and Centor score of 4 are associated with antibiotic prescription (*P* < 0.05).

Table 2 shows the distribution of the types of prescribed antibiotics. All of the prescribed antibiotics were in oral form. The most prescribed antibiotic was amoxicillin 500mg for seven days which was dispensed in 28 cases. Four out of six cases of laboratory-confirmed GAS pharyngitis were treated with antibiotics. In contrast, one case was not treated with antibiotics and one case was referred to the hospital because of unspecified complications.

### Antimicrobial Susceptibility Patterns

Beta-haemolytic streptococci were isolated from 80 (37.2%) participants. Among the 80 isolates of the beta-haemolytic streptococci, GAS was positive in 6 (2.8%) participants. All isolates were susceptible to penicillin G, ampicillin, ofloxacin, cefepime, cefotaxime, ceftriaxone, vancomycin and linezolid. However, 38.8% of beta-haemolytic streptococci (groups A, B, C, F and G) found to be resistant to tetracycline, 5% resistant to clindamycin and erythromycin resistance was 11.3%.

## Discussion

In the present study, doctors in the three primary care clinics prescribed antibiotics to 48 (22.3%) of the patients with a sore throat (*N* = 215).

From the total prescriptions in this study, 37 (77.1%) were diagnosed with pharyngitis/ tonsilitis. This shows that antibiotic prescription is associated with pharyngitis/tonsilitis (*P* < 0.005). Interestingly, overall we found only six throat swab samples were laboratory confirmed GAS, while in terms of clinical characteristics based on the Centor scoring, only three patients (1.4%) scored 4 and 8 patients (3.7%) scored 3. Antibiotics are only indicated in a patient with sore throat if it is GAS or if they have a Centor score of 4 and judiciously if they score 3. In our study, all patients who scored 4 received antibiotics and had a positive culture for GAS. However, not all patients with laboratory confirmed GAS received antibiotics in which two had positive throat swab cultures and both had a Centor score of 3. This shows that in this study, a Centor score of 4 is associated with GAS (*P* < 0.05) and antibiotic prescription (*P* = 0.001). But in terms of prescribing habit, a diagnosis of pharyngitis/tonsilitis correlates with the decision to prescribe, although most of these cases do not fulfil the clinical criteria of GAS pharyngitis as we found 42 patients who were prescribed antibiotics (87.5%) had a Centor score of 0–2. Studies have shown with a sore throat only 8.3% (7) of them with GAS pharyngitis, which is the only indication for antibiotics therapy (8). Therefore, the over prescription rate in this study was 91.7% (9, 10). For most patients with respiratory tract infection, antibiotic use is unnecessary and these results suggest that antibiotics were not clinically indicated, therefore overused in the three primary care clinics in Malaysia. Excessive or suboptimal use of medicine in general and antibiotics in particular, is a worldwide concern (16, 17).

The antibiotic prescription rate in this study was lower than the rate in a study at public primary care clinics which was carried out in Malaysia, where the antibiotic prescription rate was 34.1% among patients with a sore throat (18). It was comparable to a study conducted in Malaysia where 21.1% of antibiotics prescribed for upper respiratory tract infections (19). A study was done by Hicks et al. (20) in the United States of America has reported that the rate of prescribed antibiotics to adults with a sore throat was 73.7%. However, another study about the variations of prescribed antibiotics in the European Union among adults with a sore throat has shown that the prescribed antibiotics rates were lower than this study in the Netherlands (8.9%) and Sweden (13.5%). In comparison, it was close to a study in Belgium (26.7%) (21). Although the rate of the prescribed antibiotics in this study is lower than the rates in other studies but it exceeds the expected prevalence of GAS among adults.

In this study, the majority of prescribed antibiotics were amoxicillin and erythromycin. Oral amoxicillin 500 mg, oral co-amoxiclav 625 mg and oral cephalexin 500 mg were prescribed for the patients with GAS pharyngitis for 5 days–7 days by the physicians in the primary care clinics. Meanwhile, 44 patients diagnosed with non-GAS were treated with oral EES 800 mg, oral EES 400 mg, oral amoxicillin 500 mg, oral amoxicillin 250 mg, oral cefuroxime axetil 125 mg, oral cephalexin 500 mg and oral cloxacillin 500 mg for 5 days–7 days. The National Antibiotic Guideline in Malaysia has recommended amoxicillin 500 mg or phenoxymethylpenicillin 500 mg for 10 days to treat pharyngitis that are caused by GAS; however, the alternative therapy is a single dose of benzathine penicillin, azithromycin 500 mg for 5 days or clindamycin 300 mg–450 mg for 10 days to patients who allergic to penicillin (22). Other studies have shown that oral penicillin V for 10 days or a single dose of parenteral penicillin G was the first line of antibiotics to treat GAS pharyngitis and it was reduced the duration of the symptom (23, 24). In addition, another study has reported that penicillin antibiotic treatment within 9 days was sufficient to prevent rheumatic fever (25). In contrast, other studies have proved that treatment of GAS pharyngitis by amoxicillin, erythromycin, oral cephalosporins and clindamycin for 7 days was sufficient to reduce the symptoms (26, 27). Therefore, this study has found that the physicians in the three clinics did not follow the National Antibiotic Guideline in Malaysia to prescribe antibiotics to adult patients with a sore throat.

In this study, among the 80 isolates of beta-haemolytic streptococci (groups A, B, C, F and G) which collected from the throat of adults with a sore throat in the three primary care clinics, there were no strains resistant to penicillin, ampicillin, ofloxacin, cefepime, cefotaxime, ceftriaxone, linezolid and vancomycin and there were strains resistant to tetracycline, erythromycin and clindamycin. Penicillin or ampicillin is the first line of antibiotic which is recommended to treat GAS pharyngitis by international and local guidelines (28). The present study confirmed that the resistance to penicillin or ampicillin has not identified in beta-haemolytic streptococcal (groups A, B, C, F and G) strains, thus far (29, 30), and even study in Sweden about penicillin tolerance in GAS has found five strains from cases cause treatment failures in pharyngitis seems doubtful (31). Beta-haemolytic streptococcal (groups A, B, C, F and G) isolates were found to be susceptible to ofloxacin, cefepime, cefotaxime, ceftriaxone, linezolid and vancomycin. Previous studies have confirmed the results of this study, where the results obtained have shown the similar activity of ofloxacin, cefepime, cefotaxime, ceftriaxone, linezolid and vancomycin against beta-haemolytic streptococci (groups A, B, C, F and G) (32–34). However, a study comparing the susceptibility of clinical group A, B, C, F and G beta-haemolytic streptococcal isolates for 24 antimicrobial drugs found two group C streptococcal strains (1.9%) to be resistant to ofloxacin (35). In addition, a study carried out among 177 patients with pharyngitis in Iraq has reported one group F streptococcal isolate to be resistant to vancomycin (36). However, Clinical and Laboratory Standards Institute (CLSI) documents have reported that the isolates with reduced susceptibility to vancomycin cannot be differentiated from susceptible isolates by disk diffusion test (15). Clindamycin and macrolides are available and are used as an alternative therapy to treat streptococcal pharyngitis for patients allergic to penicillin (37). This study had detected low level resistance to clindamycin and erythromycin where 5% of beta-haemolytic streptococci (groups A, B, C, F and G) isolates were resistant to clindamycin and 11.3% were resistant to erythromycin. This result correlates with a study in Northern Spain, which has reported a low rate of erythromycin and clindamycin resistance where the rate of erythromycin resistance was 9.3 % and clindamycin resistance was 1.7% (38). Tetracycline is not recommended as a treatment for streptococcal pharyngitis but it is a broad spectrum antibiotic that can be used to treat a variety of bacterial infections, which means a high total level of consumption (39). The resistance to tetracycline has been widespread among beta-haemolytic streptococci (40). This study confirmed what other studies had reported about the high resistance of beta-haemolytic streptococci (groups A, B, C, F and G) to tetracycline especially GAS and group B streptococci, where the resistance of betahaemolytic streptococci was 38.8% (35). As another study in Thailand has reported that 52% of beta-haemolytic streptococci were resistant to tetracycline (41). In addition, a high tetracycline resistance rate of beta-haemolytic streptococci (groups A, B, C, F and G) was reported in other studies performed in India (73%), Germany (74.5%) and France (88.1%) (42–44). In contrast to this study, low levels of tetracycline resistance among beta-haemolytic streptococcal isolates were reported in Spain (7.3%) and in Sweden (1.3%) (45, 46). In Japan, the resistance rate of beta-haemolytic streptococci (groups A, B, C, F and G) to tetracycline was declined from 61.2% in 1981 to 20% in 1990 (47). Therefore, tetracycline is not recommended as a treatment for streptococcal pharyngitis.

Erythromycin resistance rate for GAS in Malaysia remained constant between 4.3% and 5.7% over the past 5 years and between 0.8% and 2.2% for ampicillin. Whereas tetracycline resistance has been recorded to be between 55.6% and 58.4% in the same period (11, 12).

This study has some limitations, the definitions of pharyngitis, tonsillitis and URTI, which used in this study were intentionally broad because diagnoses were not necessarily accurately recorded in the medical record. The definition of pharyngitis, tonsillitis and URTI would have captured almost all ‘true’ URTI but may have included some patients presenting with undifferentiated cough, runny nose, sore throat, injected throat and enlarged tonsils that may have turned out not to be a URTI. In addition, this study was limited to three primary clinics only from one area.

## Conclusion

This study has confirmed that antibiotics are frequently prescribed in the Malaysian primary care settings and antibiotic prescribing rates for URTI is high. Excessive and inappropriate antibiotic use in Malaysian primary care setting highlights the need for more concerted interventions targeting prescribers as well as the general public. Therefore, the antibiotic prescribing rates in Malaysia may still be a cause for concern as the high prescribing rate may cause increasing antibiotic resistance in the future. There is a need to develop educational materials and training programmes for health care physicians regarding the guidelines of antimicrobial prescribing, antibiotic resistance, clinical diagnosis, clinical scoring and the effects of unnecessary antibiotic prescription. Improvement strategies should focus on reducing inappropriate prescribing. Penicillin and ampicillin might serve as the preferred antibiotics to treat pharyngitis that are caused by beta-haemolytic streptococci.

## Figures and Tables

**Table 1:** Characteristics of patients prescribed with antibiotics

Variables	Antibiotic prescribed (*n* = 48) *n* (%)	*P*-value
Age group (years old)		
18–28	23 (47.9)	0.103^c^
29–39	15 (31.2)	
40–50	6 (12.5)	
51–60	3 (6.3)	
≥ 61	1 (2.1)	
Gender		
Male	25 (52.1)	0.121
Female	23 (47.9)	
Ethnicity		
Malay	38 (79.2)	0.033^c^*
Indian	0 (0.0)	
Chinese	10 (20.8)	
Others	0 (0.0)	
Smoking status		
Smoker	14 (29.2)	0.033*
Non-smoker	34 (70.8)	
Clinical manifestations		
Cough	42 (87.5)	0.385^c^
Rhinorrhea	37 (77.1)	0.690
Swollen anterior cervical lymph	18 (37.5)	0.008*
Fever ≥ 38 °C	6 (12.5)	0.088
Tonsillar swelling or exudates	25 (52.1)	0.001*
Fever ≥ 37.5 °C	9 (18.8)	0.181
Diagnosis		
Pharyngitis/tonsillitis	37 (77.1)	0.008*
URTI	4 (8.2)	0.077
Coryza	3 (6.3)	0.294
Pneumonia	0 (0.0)	1.000^c^
Others	4 (8.4)	0.015*
Unspecified cases^a^	0 (0.0)	< 0.001^c^*
Throat swab results		
GAS	4 (8.3)	0.023^c^*
Non-GAS^b^	44 (91.7)	
Centor score		
Score 0–1	36 (75.0)	0.048*
Score 2	6 (12.5)	0.560
Score 3	3 (6.3)	0.294
Score 4	3 (6.3)	0.001^c^

* Notes:

a Either cases have not identified the diagnosis or have identified symptoms only;

b Cases which were negative for GAS test;

c Fisher’s exact test;

* *P* < 0.05

**Table 2:** Distribution of the types of prescribed antibiotics

Prescribed antibiotics	*n* (%)
Oral erythromycin (EES) 800 mg	5 (10.4)
Oral erythromycin (EES) 400 mg	7 (14.6)
Oral amoxicillin 500 mg	28 (58.3)
Oral amoxicillin 250 mg	1 (2.1)
Oral co-amoxiclav 625 mg	1 (2.1)
Oral cefuroxime 125 mg	1 (2.1)
Oral cephalexin 500 mg	4 (8.3)
Oral cloxacillin 500 mg	1 (2.1)
Total	48 (100)
